# Three-Dimensional Geometric Morphometric Analysis of Fossil Canid Mandibles and Skulls

**DOI:** 10.1038/s41598-017-10232-1

**Published:** 2017-08-25

**Authors:** Abby Grace Drake, Michael Coquerelle, Pavel A. Kosintsev, Olga P. Bachura, Mikhail Sablin, Andrei V. Gusev, Lacey S. Fleming, Robert J. Losey

**Affiliations:** 1 Department of Ecology and Evolutionary Biology, Cornell University, 215 Tower Rd, Ithaca, NY 14853 USA; 2 Faculty of Medicine, Department of Stomatology, University Rey Juan Carlos, 28922 Alcorcon, Spain; 3 Palaeoecology Laboratory, Institute of Plant and Animal Ecology, Ural Division of the Russian Academy of Science, 8 Marta Street, #202, Ekaterinburg, 620144 Russia; 4 Zoological Institute RAS, Universitetskaya nab. 1, Saint-Petersburg, 199034 Russia; 5 Artic Research Center, Yamal-Nenets Autonomous District, Respublika St. 73, office 606, Salekhard, 629008 Russia; 6 Department of Anthropology, 13-8 Tory Building, University of Alberta, Edmonton, Alberta T6G 2H4 Canada

## Abstract

Much of the fossil record for dogs consists of mandibles. However, can fossil canid mandibles be reliably identified as dogs or wolves? 3D geometric morphometric analysis correctly classifies 99.5% of the modern dog and wolf mandibles. However, only 4 of 26 Ust’-Polui fossil mandibles, a Russian Arctic site occupied from 250BCE to 150CE, were identified as dogs and none of the 20 Ivolgin mandibles, an Iron Age site in southern Russia, were identified as dogs. Three of the Ust’-Polui mandibles and 8 of the Ivolgin mandibles were identified as wolves. In contrast, all 12 Ivolgin skulls and 5 Ust’-Polui skulls were clearly identified as dogs. Only the classification of the UP6571 skull as a dog (Dog Posterior Probability = 1.0) was not supported by the typical probability. Other evidence indicates these canids were domesticated: they were located within human dwellings, remains at both sites have butchery marks indicating that they were consumed, and isotope analysis of canid and human remains from Ust’-Polui demonstrate that both were consuming freshwater protein; indicating that the humans were feeding the canids. Our results demonstrate that the mandible may not evolve as rapidly as the cranium and the mandible is not reliable for identifying early dog fossils.

## Introduction

The date and location of dog domestication is a contentious issue whether the evidence being considered is genetic or morphological^1–14^. Previous research on canid fossil mandibles and skulls has employed Euclidean distances for identification^1, 3, 7^. We conducted a three-dimensional geometric morphometric analysis of fossil mandibles and skulls from Ust’-Polui, Ivolgin, and Alaska to determine whether these are dog or wolf fossils^6, 15–19^. The Ust’-Polui and Ivolgin sites date to the late Holocene, post-dating the advent of dog domestication by millennia^16–19^. The Ust’-Polui archaeological site is in Salekhard, Russia, in the Arctic (66.5501°N, 66.6028°E). Ust’-Polui has produced thousands of artifacts and faunal remains, including disarticulated skeletal elements from over 100 canids, nearly all of which were originally identified as dogs based on their relative small sizes compared to Arctic wolves^18, 19^. At least two of the canid skeletons found at this site were fully articulated burials (these canids were not available for the present analysis). Radiocarbon dating and dendrochronology indicate this fortified site was occupied from ~250BCE to 150CE by foragers^18, 19^. The Ivolgin site is in the steppe region of southern Russia near Ulan-Ude (51.7630°N, 107.47346°E). Ivolgin consisted of a series of earth ramparts and a wood stockade that surrounded over 50 wooden dwellings^16, 17^. This town was probably occupied by the Xiongnu, a confederation of Iron Age pastoral groups, from ~300BCE to 200CE^16, 17^. Scattered faunal remains were found throughout Ivolgin, with over 90% identified as domestic animals (sheep, cattle, pig, horse, goat, camel, yak, dog). At least 59 specimens from Ivolgin were previously identified as dogs based on size comparisons with southern Siberian wolves^16^. The only wild canid identified was fox^16^. At both Ust’-Polui and Ivolgin, some of the canid remains display butchery marks, indicating they were consumed by people. Additionally, we analyzed carbon-dated late Pleistocene canid fossils from Alaskan permafrost deposits that were all genetically classified as wolves^20^. Finally, Alaskan canids carbon-dated near 1600CE and genetically classified as dogs also were analyzed^21^. These two sets of Alaskan canid fossils were included to determine whether genetic identifications correspond with those based on morphology.

Traditional morphometric analyses of fossil canid mandibles and skulls aimed at identifying dogs depend mostly on one-dimensional measures of length and width^1–5, 7^. As demonstrated in our previous analysis of the canid skull^6^, distance measurements are problematic for multiple reasons including: autocorrelation, isometry, overlap between dogs and wolves (making identification impossible), spurious correlations in multivariate analyses, and non-normal distributions^22^. Capturing 3D coordinates from mandibles and skulls provides a more accurate representation of their inherent 3D shapes than one-dimensional distances and ratios (Supplementary Figure S1)^23, 24^. Geometric morphometric methods (GMM) are widely recognized as powerful and sophisticated diagnostic tools for investigating biological shape^6, 15^. Procrustes superimposition of the coordinate configurations removes information related to size by scaling all configurations to the same centroid size while translating and rotating the landmark configurations using a least-squares fit of homologous landmarks^24, 25^. Thus, Procrustes coordinates only contain information pertaining to shape, having removed information related to size, position, and rotation^24, 25^. Here we re-analyze the canid skulls and mandibles from the Ust’-Polui, Ivolgin, and Alaska sites using 3D GMM to assess whether they can be accurately identified as dogs or wolves. We compared the fossil mandibles and skulls to a large dataset of modern mesaticephalic (wolf-like) dogs and a global assemblage of both modern and fossil wolves. We hypothesize that the fossil skulls will be accurately identified as wolves or dogs; however, it remains to be seen if these fossil mandibles can be reliably categorized.

**Figure 1:**
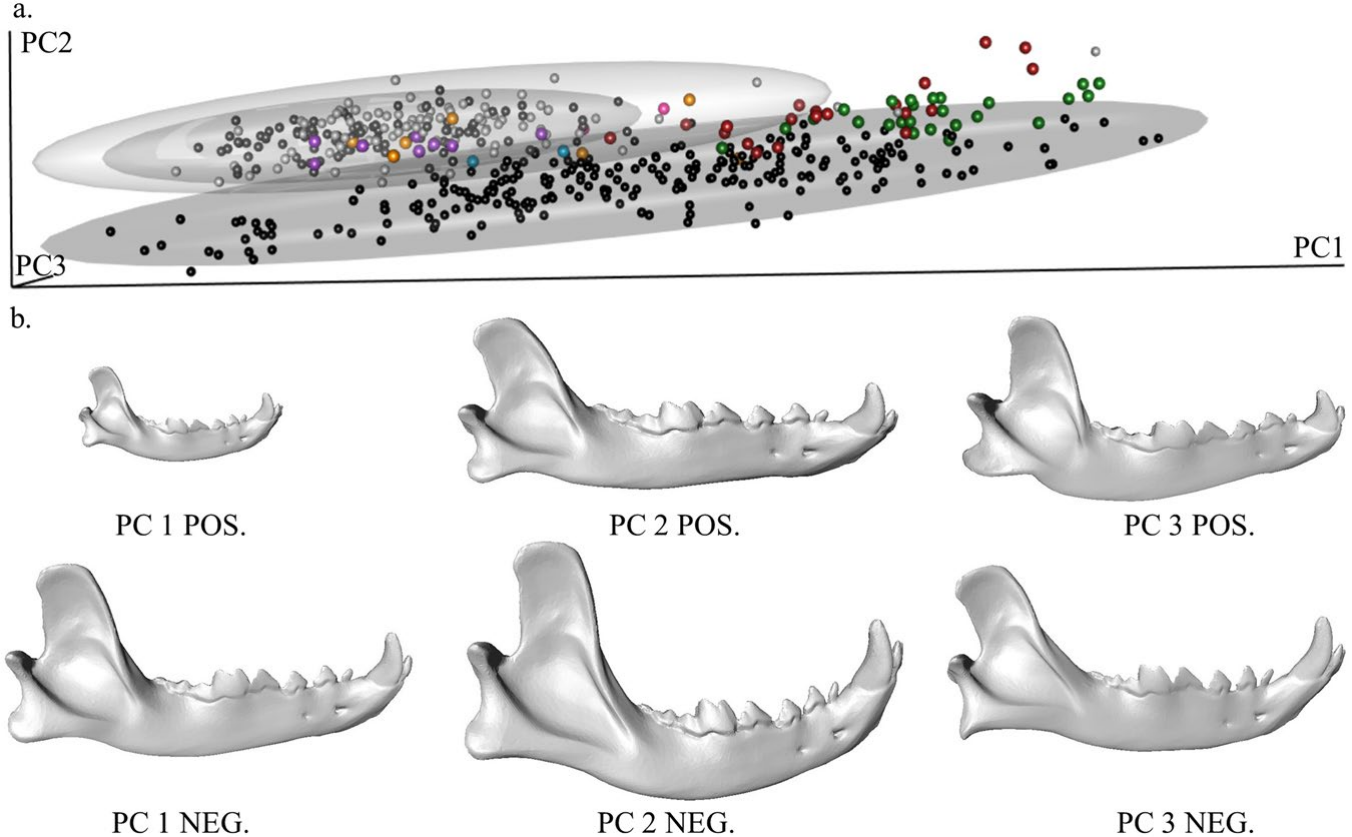
3D plot of PC1–3 mandible shape variation. Black: dogs, dark grey: Alaskan wolves, light grey: European wolves, dark red: Ivolgin fossils, green: Ust’-Polui fossils, purple: Pleistocene Alaskan wolves, cyan: 1600CE fossil dogs, orange: unknown Alaskan fossil canids, pink: 1600CE fossil wolf.

The form-space principal components analysis (PCA) revealed that modern dog and wolf mandibles are separated within the first three principal components, which account for 92.3% of the total mandible form-space variance (Fig. 1a). PC1 captures static allometry and is associated with overall size variation (r_PC1_ = 0.99, P < 0.001) from the large, robust jaws of wolves and dog breeds such as German Shepherds to the smaller mandibles of breeds like the Fox Terrier (Fig. 1b and Supplementary Video S1). Wolves have significantly larger mandibles than dogs (P < 0.001; permutation test, n = 10,000). However, due to their considerable variation in size, dogs overlap with wolves along PC1 (Fig. 1a). Dogs diverge from wolves along PCs 2 and 3, which are both independent of size (r_PC2_ = 0.002, r_PC3_ = 0.003). The curvature of the mandible distinguishes dogs from wolves along PC2 (Fig. 1b and Supplementary Video S2).

99.5% of modern canid mandibles were correctly identified as either dogs or wolves with 100% accuracy using a resampling procedure involving one-thousand iterations of a cross-validation Quadratic Discriminant Analysis (QDA) to ensure equal dog and wolf sample sizes, with posterior probability greater than 0.90 (Tau = 0.460, Wilks’ lambda = 0.12). Furthermore, the classifications were supported by the typical probabilities.

The fossil canid mandibles exhibit a wide variation in morphology; some are separated from both wolves and dogs at the positive end of PC1 while some are within the wolf or dog shape variation (Fig. 1a). The results from the classification procedure identified 4 of the 26 mandibles from Ust’-Polui as dogs and 3 of the mandibles as wolves (Table 1). The remaining 19 Ust’-Polui mandibles could not be statistically identified as dogs or wolves (Table 1). Within the Ivolgin population, 8 mandibles were classified as wolves and 12 were unidentifiable (Table 1). The unclassified Ust’-Polui and Ivolgin mandibles are not highly aberrant from dogs or wolves as demonstrated by their close proximity on the PCA plot (Fig. 1a). These unclassified mandibles do not share the formspace of either the dogs or the wolves. They are similar in size to smaller mesaticephalic dogs such as Fox Terriers (overlap on PC1) but the shape of their mandibles resembles the wolf mandibles (overlap on PC2). Our GMM analysis confirmed that the Alaskan Pleistocene canid mandibles are wolves (Table 1)^20^. Our results also verified that 2 of the Alaskan mandibles dated near 1600CE (AMNH30436 and AMNH70932) are dogs (Table 1)^21^. Our analysis classified the mandibles AMNH30482 and AMNH70963C as wolves despite the genetic identification by Leonard *et al*.^21^ of both specimens being dogs (Table 1). We also analyzed 7 mandibles that have not been carbon-dated nor genetically identified but were found at the same Alaskan site as the 1600CE mandibles. One of these, AMNH39381, was classified as a dog, and 6 of the mandibles were identified as wolves (Table 1).

**Table 1:** Results of the resampling procedure for the QDA of the mandibles using PCs 1–9.

Specimen	Average Ppost	Percentage of iterations for which specimen’s Ppost ≥0.90				QDA Result	Typical Probability	
	Dog	Wolf	Dog	Wolf	Ind	Group	%Typ. P ≤ 0.05	Group
UP 1	0.45	0.55	0.50	6.50	93.00	Ind.	60.10	Ind.
UP 2	0.33	0.67	1.80	24.70	73.50	Ind.	100.00	Ind.
UP 3	0.00	1.00	0.00	100.00	0.00	Wolf	100.00	Ind.
UP 4	0.01	0.99	0.00	99.30	0.70	Wolf	100.00	Ind.
UP 5	1.00	0.00	100.00	0.00	0.00	Dog	0.00	Dog
UP 6	0.04	0.96	0.00	91.50	8.50	Ind.	0.00	Wolf
UP 7	1.00	0.00	99.80	0.00	0.20	Dog	1.20	Dog
UP 8	0.39	0.61	2.40	20.20	77.40	Ind.	100.00	Ind.
UP 9	0.97	0.03	93.90	0.40	5.70	Ind.	100.00	Ind.
UP 10	0.03	0.97	0.00	95.30	4.70	Wolf	100.00	Ind.
UP 11	0.00	1.00	0.00	100.00	0.00	Wolf	0.00	Wolf
UP 12	0.79	0.21	43.20	0.60	56.20	Ind.	52.20	Ind.
UP 13	1.00	0.00	100.00	0.00	0.00	Dog	97.60	Ind.
UP 14	1.00	0.00	100.00	0.00	0.00	Dog	4.90	Dog
UP 15	1.00	0.00	100.00	0.00	0.00	Dog	100.00	Ind.
UP 16	0.15	0.85	0.00	40.50	59.50	Ind.	0.10	Ind.
UP 17	0.01	0.99	0.00	100.00	0.00	Wolf	0.00	Wolf
UP 18	0.62	0.38	2.90	0.50	96.60	Ind.	0.00	Ind.
UP 19	0.13	0.87	0.10	64.40	35.50	Ind.	100.00	Ind.
UP 20	1.00	0.00	100.00	0.00	0.00	Dog	98.90	Ind.
UP 21	0.39	0.61	5.30	21.70	73.00	Ind.	100.00	Ind.
UP 23	1.00	0.00	100.00	0.00	0.00	Dog	1.50	Dog
UP 24	0.91	0.09	73.90	0.00	26.10	Ind.	50.00	Ind.
UP 25	0.19	0.81	0.00	43.70	56.30	Ind.	99.50	Ind.
UP 26	1.00	0.00	99.50	0.00	0.50	Dog	100.00	Ind.
IV 1	0.00	1.00	0.00	100.00	0.00	Wolf	0.00	Wolf
IV 2	0.00	1.00	0.00	100.00	0.00	Wolf	100.00	Ind.
IV 3	0.00	1.00	0.00	100.00	0.00	Wolf	100.00	Ind.
IV 4	0.87	0.13	48.20	0.00	51.80	Ind.	0.00	Ind.
IV 5	0.00	1.00	0.00	100.00	0.00	Wolf	0.00	Wolf
IV 6	0.00	1.00	0.00	100.00	0.00	Wolf	0.00	Wolf
IV 7	0.00	1.00	0.00	100.00	0.00	Wolf	100.00	Ind.
IV 8	0.00	1.00	0.00	100.00	0.00	Wolf	0.60	Wolf
IV 9	0.30	0.70	0.00	7.10	92.90	Ind.	0.00	Ind.
IV 10	0.70	0.30	10.50	0.70	88.80	Ind.	0.00	Ind.
IV 11	0.00	1.00	0.00	100.00	0.00	Wolf	0.50	Wolf
IV 12	0.01	0.99	0.00	100.00	0.00	Wolf	0.00	Wolf
IV 13	0.00	1.00	0.00	100.00	0.00	Wolf	0.10	Wolf
IV 14	0.00	1.00	0.00	100.00	0.00	Wolf	0.00	Wolf
IV 15	0.00	1.00	0.00	100.00	0.00	Wolf	100.00	Ind.
IV 16	0.00	1.00	0.00	100.00	0.00	Wolf	100.00	Ind.
IV 17	0.00	1.00	0.00	100.00	0.00	Wolf	100.00	Ind.
IV 18	0.00	1.00	0.00	100.00	0.00	Wolf	100.00	Ind.
IV 19	0.26	0.74	0.00	30.20	69.80	Ind.	100.00	Ind.
IV 20	0.21	0.79	0.00	34.60	65.40	Ind.	47.20	Ind.
**Fossil canids from Alaska:**								
AMNH 30436^**^	0.97	0.03	98.30	0.00	1.70	Dog	0.00	Dog
AMNH 30474	0.00	1.00	0.00	100.00	0.00	Wolf	0.00	Wolf
AMNH 30482^**^	0.01	0.99	0.00	100.00	0.00	Wolf	0.00	Wolf
AMNH 39381	1.00	0.00	100.00	0.00	0.00	Dog	0.00	Dog
AMNH 67168^*^	0.00	1.00	0.00	100.00	0.00	Wolf	0.00	Wolf
AMNH 67169^*^	0.00	1.00	0.00	100.00	0.00	Wolf	0.00	Wolf
AMNH 67179^*^	0.00	1.00	0.00	100.00	0.00	Wolf	0.00	Wolf
AMNH 67202^*^	0.00	1.00	0.00	100.00	0.00	Wolf	0.00	Wolf
AMNH 67224^*^	0.00	1.00	0.00	100.00	0.00	Wolf	0.00	Wolf
AMNH 67228^*^	0.00	1.00	0.00	100.00	0.00	Wolf	0.00	Wolf
AMNH 67242	0.00	1.00	0.00	100.00	0.00	Wolf	0.00	Wolf
AMNH 70932^**^	1.00	0.00	100.00	0.00	0.00	Dog	0.00	Dog
AMNH 70944^*^	0.00	1.00	0.00	100.00	0.00	Wolf	0.00	Wolf
AMNH 70958^*^	0.00	1.00	0.00	100.00	0.00	Wolf	0.00	Wolf
AMNH 97104	0.98	0.02	100.00	0.00	0.00	Dog	0.00	Wolf
AMNH 97105	0.00	1.00	0.00	100.00	0.00	Wolf	0.00	Wolf
AMNH 70963C^**^	1.00	0.00	100.00	0.00	0.00	Dog	0.00	Wolf
AMNH AINS825	0.00	1.00	0.00	100.00	0.00	Wolf	0.00	Wolf
AMNH AINS840	0.00	1.00	0.00	100.00	0.00	Wolf	0.00	Wolf

*Specimen identified as a wolf by Leonard *et al*.^20^. **Specimen identified as a dog by Leonard *et al*.^21^.

**Figure 2:**
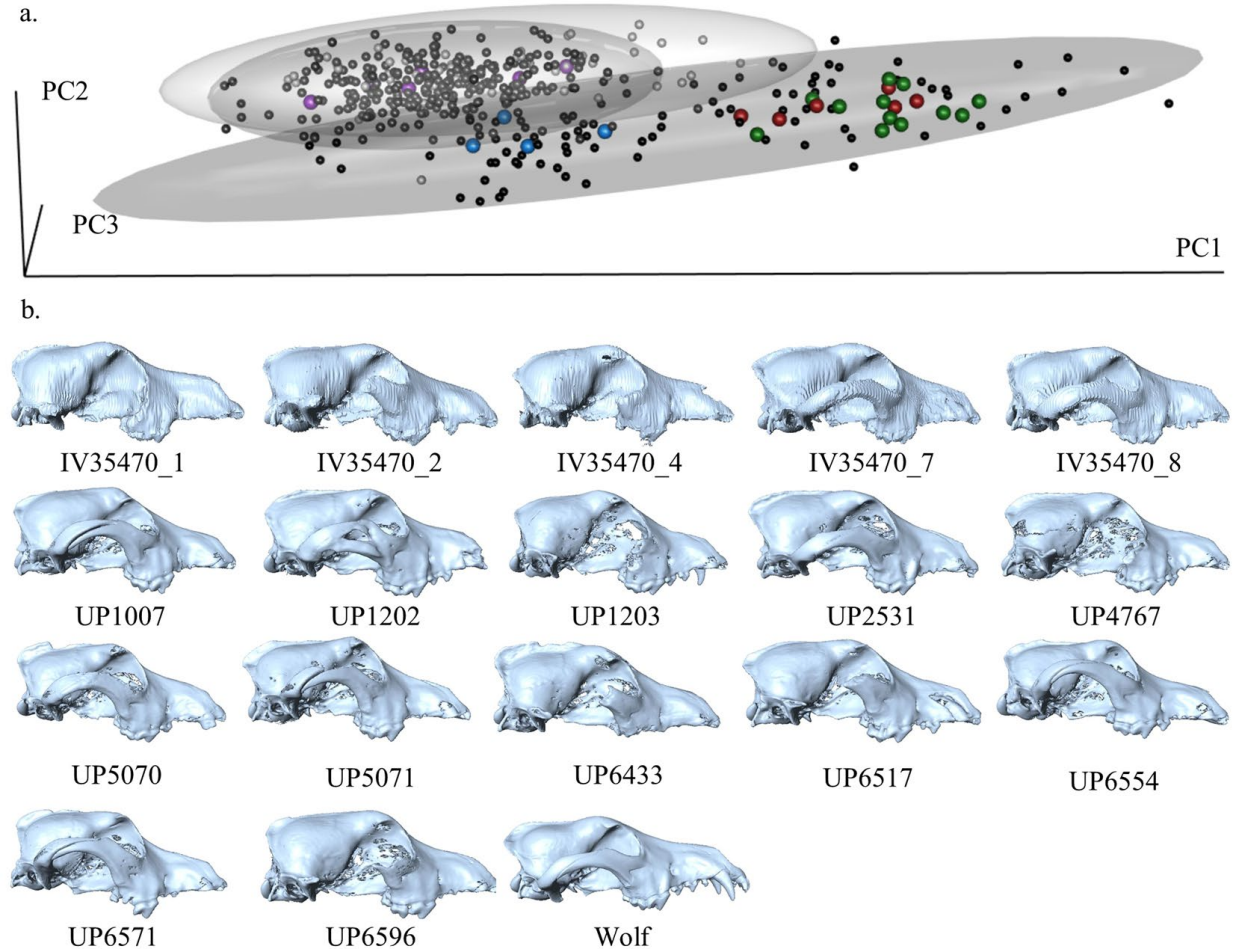
3D plot of PC1–3 skull shape variation. Black: dogs, dark grey: Alaskan wolves, light grey: European wolves, dark red: Ivolgin fossils, green: Ust’-Polui fossils, purple: Pleistocene Alaskan wolves, cyan: 1600CE fossil dogs.

We conducted a separate three-dimensional geometric morphometric analysis of the fossil skulls from Ivolgin, Ust’-Polui, and Alaska and compared them to modern mesaticephalic (wolf-like) dogs, ancient dogs, and the global assemblage of both modern and fossil wolves from our previous study^6^. The form-space PCA clearly shows the fossil canid skulls from Ust’-Polui and Ivolgin all lie within dog cranial shape variation and are clearly separated from wolf cranial shape variation within the first three principal components which account for 89.7% of the total skull form variance (Fig. 2a). The Ust’-Polui and Ivolgin fossil skulls all share with modern dogs forward-facing orbits and almost all display a pronounced angle between the forehead and the muzzle, a distinguishing feature of dog skulls (Fig. 2b)^6^. The results from the classification procedure identify all fossil skulls as dogs according to both the posterior and typical probabilities in 99.6% of the 1,000 resampling runs (Table 2). Only the classification of UP6571 as a dog via posterior probability (Dog Posterior Probability = 1.0) was not supported by the typical probability in more than 95% of the resampling runs (Table 2). The late Pleistocene Alaskan fossil canid skulls were all confirmed to be wolves except for AMNH30433 (wolf posterior probability = 0.87) and AMNH67157 (wolf posterior probability = 0.66). The Alaskan fossil canids from 1600CE were all identified as dogs in our analysis, corroborating the genetic identification of these specimens as dogs^21^.

**Table 2:** Results of the resampling procedure for the QDA of the skulls using PCs 1-6-25.8.

Specimen	Average Ppost	Percentage of iterations for which specimen’s Ppost ≥0.90				QDA Result	Typical Probability	
	Dog	Wolf	Dog	Wolf	Ind	Group	%Typ. P ≤ 0.05	Group
UP 1007	1.00	0.00	100.00	0.00	0.00	Dog	0.00	Dog
UP 1202	1.00	0.00	100.00	0.00	0.00	Dog	0.00	Dog
UP 1203	1.00	0.00	100.00	0.00	0.00	Dog	0.00	Dog
UP 2531	1.00	0.00	100.00	0.00	0.00	Dog	0.00	Dog
UP 4767	1.00	0.00	100.00	0.00	0.00	Dog	0.40	Dog
UP 5070	1.00	0.00	100.00	0.00	0.00	Dog	0.00	Dog
UP 5071	1.00	0.00	100.00	0.00	0.00	Dog	0.00	Dog
UP 6433	1.00	0.00	100.00	0.00	0.00	Dog	0.00	Dog
UP 6517	1.00	0.00	100.00	0.00	0.00	Dog	0.00	Dog
UP 6554	1.00	0.00	100.00	0.00	0.00	Dog	0.00	Dog
UP 6571	1.00	0.00	100.00	0.00	0.00	Dog	44.00	Ind.
UP 6596	1.00	0.00	100.00	0.00	0.00	Dog	0.00	Dog
IV 35470_1	1.00	0.00	100.00	0.00	0.00	Dog	0.00	Dog
IV 35470_2	1.00	0.00	100.00	0.00	0.00	Dog	0.00	Dog
IV 35470_4	1.00	0.00	100.00	0.00	0.00	Dog	0.00	Dog
IV 35470_5	1.00	0.00	100.00	0.00	0.00	Dog	0.00	Dog
IV 35470_7	1.00	0.00	100.00	0.00	0.00	Dog	0.00	Dog
IV 35470_8	1.00	0.00	100.00	0.00	0.00	Dog	2.40	Dog
**Fossil canids from Alaska:**								
AMNH 30431^*^	0.00	1.00	0.00	99.9	0.10	Wolf	0.00	Wolf
AMNH 30433^*^	0.13	0.87	0.20	60.5	39.30	Ind.	0.00	Ind.
AMNH 30450^*^	0.00	1.00	0.00	100.0	0.00	Wolf	0.00	Wolf
AMNH 67157^*^	0.34	0.66	2.10	16.6	81.30	Ind.	0.00	Ind.
AMNH 67163^*^	0.00	1.00	0.00	100.0	0.00	Wolf	0.00	Wolf
AMNH 97079^*^	0.02	0.98	0.00	99.3	0.70	Wolf	0.00	Wolf
AMNH 30435^**^	1.00	0.00	100.0	0.00	0.00	Dog	0.00	Dog
AMNH 30436^**^	1.00	0.00	100.0	0.00	0.00	Dog	0.00	Dog
AMNH 67155a^**^	1.00	0.00	100.0	0.00	0.00	Dog	0.00	Dog
AMNH 70932^**^	1.00	0.00	99.90	0.00	0.10	Dog	0.00	Dog

Surprisingly, only 15% of the mandible specimens from the Ust’-Polui site were classified as dogs despite the very high accuracy of this procedure in correctly assigning known specimens to their group. In addition, 69% of the mandible specimens from Ust’-Polui and 60% of the mandible specimens from Ivolgin were unclassified as either dogs or wolves. The unclassified mandibles are found outside the wolf mandible morphospace because they are smaller than the wolves and outside the dog mandible morphospace because of their wolf-like shape. However, all but one of the skull specimens from both sites were identified as dogs. Interestingly, we saw a similar pattern in the fossil canids from Alaska. Although 4 of 1600CE Alaskan fossil mandibles had been genetically identified as dogs^21^, our analysis only classified 2 of these mandibles as dogs (Table 1). Furthermore, the canids at Ust’-Polui and Ivolgin are suspected to be domestic dogs based on other criteria. Those at Ivolgin were found in association with remains of many other domestic fauna and in a fortified town occupied by a historically-documented pastoral society, some inside houses and waste pits. Stable carbon and nitrogen isotope analysis of bone collagen from 44 Ust’-Polui canid specimens, including 10 crania and 34 right scapulae, indicated these individuals have very negative δ^13^C values (mean δ^13^C = −25.8‰, s.d. = 0.8) and elevated δ^15^N values (mean δ^15^N = 13.9‰, s.d. = 0.8) (Supplementary Table S1; For isotope analysis methods see Supplementary Information). The canids’ isotope values are similar to those of two humans buried at Ust’-Polui (mean δ^13^C = −25.3‰, s.d. = 1.25; mean δ^15^N = 16.9‰, s.d. = 0.4; Supplementary Figure S2). Terrestrial herbivores such as reindeer (*Rangifer tarandus*) and elk (*Alces alces*) at the site have far more positive δ^13^C values (mean δ^13^C = −20.0‰, s.d. = 1.03), and much lower δ^15^N values (mean δ^15^N = 5.6‰, s.d. = 2.1). Bone collagen δ^15^N values show enrichment of 3–5‰ along the food chain, providing an indication of trophic level^26, 27^. The canids and humans at Ust’-Polui, all with δ^15^N values above 12‰, were regularly consuming food items with higher δ^15^N values than those of these large-bodied terrestrial herbivores. In the Arctic, such elevated δ^15^N values appear more consistent with dietary reliance on freshwater or marine fauna. Neither freshwater fish or marine mammals are well represented in our isotope data, but other studies show that Arctic freshwater fish have far more negative δ^13^C values than marine mammals and fish, ranging from around −30‰ to −20‰, with the two groups of marine fauna often having more positive values than terrestrial ungulates^28–35^. Offsets in δ^13^C between prey collagen and predator collagen are around 1‰^26, 36^. The very negative δ^13^C values for the canids and humans at Ust’-Polui suggest both were regularly consuming freshwater fish; remains of such fish are highly abundant at the site^19^. Such dietary patterns are inconsistent with those of wolves living in the Arctic, where water bodies are frozen for much of the year^37, 38^, but are consistent with people partially provisioning dogs with their own food items, a historically well-documented practice in many areas^39^.

The lack of consistent identification of the mandibles as either dogs or wolves could indicate several things. The mandibles seem unlikely to be from small wolves or hybrids because neither wolf nor hybrid crania were found at either site; all of the skulls from both sites were clearly identified as dogs. It also seems improbable that the mandibles are from some other type of canid, as one would expect their crania also to be present at these sites, both of which have been extensively excavated. Perhaps most telling, even some of the Late Holocene mandibles from Alaska were not morphologically identified, despite the identification of the skulls as dogs as well as their confirmation as dogs from genetic information^21^.

Contextual and dietary information from both Ivolgin and Ust’-Polui provide supporting evidence for the presence of dogs at these two sites. The mandibles from Ivolgin were found with remains other domestic animals within a fortified town. Analysis of the canid skulls at both Ust’-Polui and Ivolgin confirm that numerous dogs are present at both locations but failed to show the presence of wolves. Further, there are butchery marks on the canid remains at both of these sites, and the isotope analysis indicates that the canids at Ust’-Polui had similarly structured diets as the humans buried there, and these diets included freshwater fish.

Overall, these results indicate that the rate of evolutionary modification of the dog mandible may not keep pace with cranial shape change, and that the variation in mandible shape that differentiates modern dogs and wolves mostly emerged relatively late in the domestication process, perhaps with the advent of modern intensive breeding. Fossil mandibles from even Late Holocene dogs may be mistakenly classified as small wild canids and should not be relied on as the only evidence for specimen identification. Future studies comparing shape variation in canid skulls and mandibles may elucidate the lack of coevolution observed in our dataset.

## Methods

Ct-scans of fossil mandibles from Ivolgin and Ust’-Polui were converted into Polygon files and digitized in IDAV Landmark software^40^ all other specimens were digitized by AGD with a Microscribe digitizer. Fossils include 26 mandibles from Ust’-Polui^18, 19^, 20 from Ivolgin^16, 17^, 8 late Pleistocene canids from Alaska^20^ (AMNH: 67168, 67169, 67179, 67202, 67224, 67228, 70944, 70958), 4 canids from near 1600CE Alaska^21^ (AMNH: 30436, 30482, 70932, 70963C), and another 7 canids that are likely from around 1600 CE Alaska (but have not been carbon-dated) (AMNH: 30474, 39381, 67242, 97104, 97105, AINS825, AINS840). 37 three-dimensional coordinates were captured from the mandibles of 121 North American wolves, 85 Eurasian wolves, and 240 adult dogs (only mesaticephalic breeds) (Supplementary Figure S1). Breeds in the mandible analysis include: Afghan Hound, Airedale Terrier, Akita Inu, Alaskan Malmute, Bloodhound, Borzoi, Boxer, Bull Terrier, Chesapeake Bay Retriever, Chow Chow, Cocker Spaniel, Dalmatian, Dingo, English Bulldog, English Setter, English Springer Spaniel, Foxterrier, French Mastiff, German Shepherd, German Spaniel, Golden Retriever, Greenland Dog, Greyhound, Irish Setter, Irish Wolfhound, Jura Laufhund, Jura Laufhund St. Hubert, Labrador Retriever, Nova Scotia Duck Tolling Retriever, Pharoh Hound, Poodle, Samojede, Scottish Deerhound, Shar Pei, Siberian Husky, Tervueren, Weimeraner, Whippet, and Wolfspitz.

Ct-scans of fossil skulls from Ivolgin and Ust’-Polui, as well as the following fossil specimens: Eliseevichi MAE 447/5298 (13,905 +/− 55 YBP; Epigravettian), Goyet (31,680 +/− 250 YBP), Trou Balleux (10,110 +/− 120 YBP), Shamanka II (7,372 +/− 47 YBP), and Ust’-Belaia (6,817 +/− 63 YBP) were converted into Polygon files and digitized in IDAV Landmark software^40^ all other specimens were digitized by AGD with a Microscribe digitizer. Fossils include 12 skulls from Ust’-Polui (UP: 1007, 1202, 1203, 2531, 4767, 5070, 5071, 6433, 6517, 6554, 6571, 6596)^18, 19^, 6 fossil skulls from Ivolgin (IV: 35470_1, 35470_2, 35470_4, 35470_5, 35470_7, 35470_8)^16, 17^, 4 late Pleistocene skulls from Alaska^20^ (AMNH: 30431, 30450, 67163, 97079), and 4 skulls from near 1600CE Alaska^21^ (AMNH: 30435, 30436, 67155a, 70932). 36 three-dimensional coordinates were recorded from skulls of 258 North American wolves, 57 European wolves, and 91 adult dogs (only mesaticephalic breeds). The following fossils were included in the wolf sample: Eliseevichi MAE 447/5298, Goyet, and Trou Balleux. The following fossils were included in the dog sample: Shamanka II, Ust’-Belaia, three Egyptian mummified dogs from the Saite–Ptolemaic period, and four Neolithic and one Gallo-Roman dog from France. For more details on the specimens used in the cranial analysis please see Drake *et al*.^6^.

The majority of dog specimens are housed in the Albert Heim Collection at the Natural History Museum in Berne, Switzerland. Most of the wolf specimens are from the University of Alaska Museum in Fairbanks, Alaska. Other specimens are from the Smithsonian Institution’s National Museum of Natural History in Washington, DC, the Museum of Vertebrate Zoology at the University of California in Berkeley, California, the Natural History Museum in Berne, Switzerland, and the Zoology Department at the Natural History Museum, London.

Geometric morphometric analysis^23–25, 41^ was conducted with the R programming language. Landmark software was used to warp a 3D Ct-scan of a wolf mandible and a wolf skull to the average shape of the known dog and wolf specimens and then warped along the PC axes^40^. Many of the methods used in this analysis are similar to those used in Drake *et al*.^6^. Here we detail any differences in methodology.

We used a resampling procedure to balance the sample sizes of the wolf and dog groups. A test developed by Anderson determined that eigenvalues from PC 9 onwards were nearly equal and therefore not useful for our analysis. We ran 1,000 iterations of the resampling procedure and in each round we used the Anderson test to determine whether the first 9 PCs were useful. If they were not, we eliminated that round and ran the resampling procedure again. For each successful round of resampling we used the 9 PCs to conduct a quantitative discriminant function analysis (QDFA) of dogs and wolves. The posterior probability and typical probabilities were calculated in the same manner as in Drake *et al*.^6^. For the analysis of the skulls we followed the same procedure, using PCs 1–6.

### Data Availability

The datasets analysed during the current study are available from the corresponding author on reasonable request.

## Electronic supplementary material

Supplementary Video S2

Supplementary Video S3

Supplemental Information
