# Supplementary material for: Three-Dimensional Geometric Morphometric Analysis of Fossil Canid Mandibles and Skulls
Source: Sci Rep. 2017 Aug 25;7:9508. doi: 10.1038/s41598-017-10232-1 (PMC5573390; doi:10.1038/s41598-017-10232-1)
Supplement: Supplementary file 3 — Supplemental Information [file 41598_2017_10232_MOESM3_ESM.pdf]

Supplemental Information for: "Three-Dimensional Geometric Morphometric Analysis of Fossil Canid Mandibles and Skulls."

Abby Grace Drake[a*], Michael Coquerelle[b], Pavel A. Kosintsev[c], Olga P. Bachura[c], Mikhail Sablin[d], Andrei V. Gusev[e], Lacey S. Fleming[f], Robert J. Losey[f]

Isotope Methods:

The stable isotope analysis was conducted using a modified version of the Oxford sample preparation method[1]. All samples were surface cleaned with a soft brush and distilled water, and outer bone surfaces were burred off. Samples were then sonicated in several changes of distilled water. After allowing the samples to air dry, they were ground to a powder consistency in a liquid nitrogen mill. Approximately 500 mg of powder from each sample were placed in a vial with 12 mL of 1% hydrochloric acid (HCl), shaken, and allowed to demineralize. The solution was changed several times during the demineralization process, and complete demineralization of each sample was assessed visually. After demineralization, samples were centrifuged and rinsed in double-distilled water until they reached neutrality. After the demineralization process, 12 mL of .01M sodium hydroxide (NaOH) solution were added to each sample to remove humates. The vials were allowed to react, then were centrifuged and rinsed in changes of double-distilled water until they reached neutrality. Immediately following this step, another 12 mL of 1% HCl were added to sample vials. Vials were shaken and left to react, then centrifuged and rinsed with double-distilled water until neutrality. Six mL of acidulated water (pH 3) were added to each vial and shaken. The samples were then placed in a 75°C water bath and left undisturbed for 20 hours to allow the collagen to gelatinize into solution. The supranatant was filtered through a glass fiber filter paper using a 40 mm Büchner filter. Approximately 6 mL of filtrate from each sample was poured into a dual-chambered ultrafiltration vial and centrifuged until 1 mL remained in the upper chamber. This amount was pipetted into a centrifuge vial, frozen, lyophilized, then analyzed at the University of Alberta's Biogeochemical Analytical Services Laboratory (BASL). Samples were analyzed for $\delta^{13}C$ and $\delta^{15}N$ ratios using a EuroVector EuroEA3028-HT elemental analyzer coupled to a GV Instruments IsoPrime continuous-flow isotope ratio mass spectrometer. BASL used NIST 8415 whole egg powder SRM as a $\delta^{13}C$ and $\delta^{15}N$ QA/QC check

throughout analyses. Results are reported relative to international standards, VPDB for C and AIR for N. Assessment of the validity of the collagen samples involved several quality indicators: 1) percent collagen yield by weight of the original bone sample above 1%; 2) percent carbon and nitrogen by weight above ~26% for carbon and 11% for nitrogen; 3), atomic C/N ratio values between 3.1 and 3.6[2-5].

References

1. Bronk Ramsey, C., Higham, T. F. G., Bowles, A., & Hedges, R. Improvements to the pretreatment of bone at Oxford. *Radiocarbon* **46**, 155-163 (2004).
2. Ambrose, S. H. Preparation and characterization of bone and tooth collagen for isotopic analysis. *Journal of Archaeological Science* **17**, 431–451 (1990).
3. DeNiro, M. J. Postmortem preservation and alteration of in vivo bone collagen isotope ratios in relation to palaeodietary reconstruction. *Nature* **317**, 806–809 (1985).
4. DeNiro, M. J., Weiner, S. Organic matter within crystalline aggregates of hydroxyapatite: a new substrate for stable isotopic and possibly other biogeochemical analyses of bone. *Geochimica et Cosmochimica Acta* **52**, 2415-2423 (1988).
5. Van Klinken, G. J. Bone collagen quality indicators for palaeodietary and radiocarbon measurements. *Journal of Archaeological Science* **26**, 687–695 (1999).

Supplementary Figure S1: 37 Osteological landmarks recorded on the mandible.

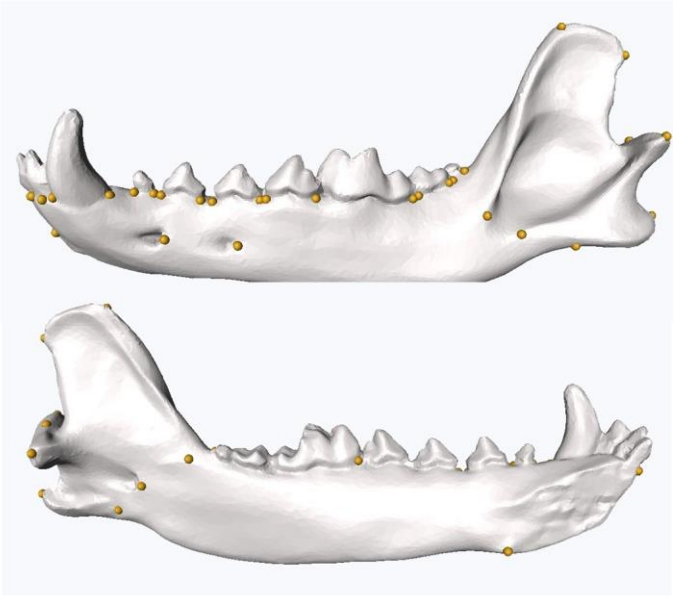

Supplementary Figure S2: Plot of stable carbon and nitrogen isotope values for humans, canids, and other fauna at Ust'-Polui. Red upside-down triangles: *Homo sapiens sapiens*, Black dots: *Canis sp.*, orange asterisk: *Alces alces*, purple square: *Alopex lagopus*, green plus: *Anas sp.*, pink triangle: *Lagopus lagopus*, blue circle: *Martes zebellina*, orange diamond: *Odobenus rosmarinus*, green diamonds: *Rangifer tarandus*

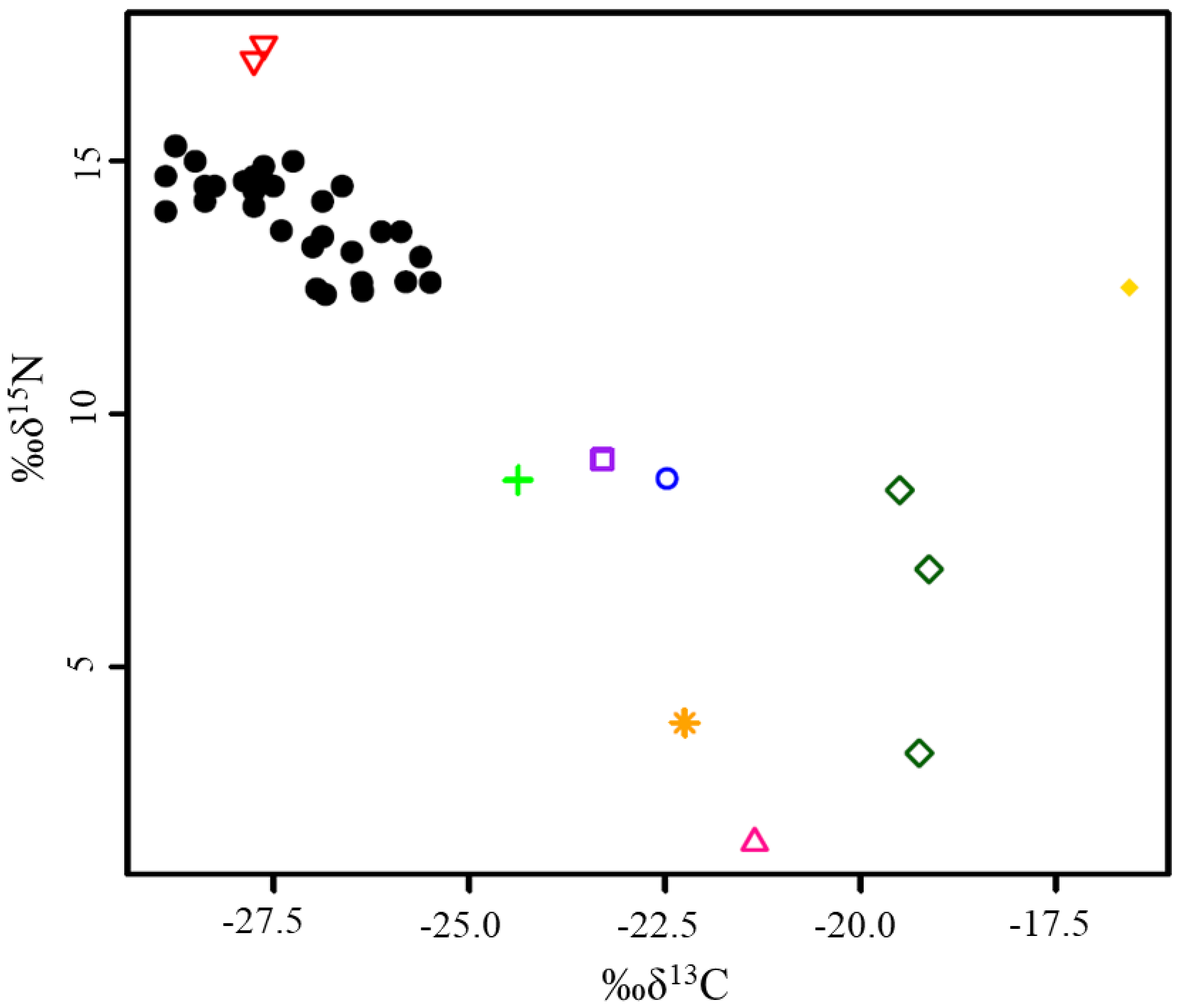

Supplementary Video S1: Video illustrating size and shape variation along form-space PC1 (static allometry) from the large jaws of wolves and breeds such as German Shepherds to smaller mandibles like the Fox Terrier.

Supplementary Video S2: Video illustrating variation along form-space PC2 from the relatively straight mandibles of wolves to the curved mandibles of dogs.

Supplementary Table S1: Stable carbon and nitrogen isotope values for human and faunal remains from Ust'-Polui.

Lab ID	Taxon	Element	Sample weight (mg)	Pre-lyophilization vial weight (mg)	Post-lyophilization vial weight (mg)	Collagen yield	δ^{13}C ‰	δ^{15}N ‰	C content (%)	N content (%)	C/N mass	C/N atomic
LPO-22F	*Alces alces*	Ph 1	500	6689.00	6695.2	1.2%	-21.8	3.9	43.6	14.8	2.9459459	3.4354326
LPO-18F	*Alopex lagopus*	costa	200	6642.7	6646.20	1.8%	-22.64	9.11	43.17	14.37	3.0041754	3.5033372
LPO-80F	*Anas* sp.	humerus	300	6638.50	6646.00	2.5%	-23.5	8.7	39.1	12.8	3.0546875	3.5622422
LPO-29F	*Canis sp.*	scapula (R)	504	9369.00	9377.10	1.6%	-26	14.5	46	16.3	2.8220859	3.2909925
LPO-30F	*Canis sp.*	scapula (R)	518	9458.30	9464.50	1.2%	-24.7	13.6	44.7	15.7	2.8471338	3.3202022
LPO-31F	*Canis sp.*	scapula (R)	510	9389.20	9453.90	12.7%	-24.4	12.6	46	16.1	2.8571429	3.3318744
LPO-32F	*Canis sp.*	scapula (R)	508	6630.50	6635.70	1.0%	-24.5	13.1	44.7	15.7	2.8471338	3.3202022

LPO-33F	*Canis sp.*	scapula (R)	514	9425	9430.60	1.1%	-26.3	14.6	43.9	15.1	2.9072848	3.3903476
LPO-34F	*Canis sp.*	scapula (R)	530	9294.30	9300.90	1.2%	-26.2	14.1	46.2	15.8	2.9240506	3.4098993
LPO-35F	*Canis sp.*	scapula (R)	524	9376.30	9384.10	1.5%	-26.2	14.4	44.3	15.2	2.9144737	3.398731
LPO-36F	*Canis sp.*	scapula (R)	532	9288.90	9294.20	1.0%	-26.1	14.6	45.6	15.6	2.9230769	3.4087638
LPO-37F	*Canis sp.*	scapula (R)	509	9428.50	9438.90	2.0%	-26.7	14.2	45.5	16.2	2.808642	3.2753148
LPO-39F	*Canis sp.*	scapula (R)	518	9434.10	9441.50	1.4%	-25.3	14.5	46	15.9	2.8930818	3.3737847
LPO-42F	*Canis sp.*	scapula (R)	512	9382.40	9391.30	1.7%	-27	15.3	46.3	16.5	2.8060606	3.2723045
LPO-44F	*Canis sp.*	scapula (R)	521	9392.10	9401.00	1.7%	-27.1	14.7	45.7	15.7	2.910828	3.3944796
LPO-47F	*Canis sp.*	scapula (R)	520	9372.10	9378.30	1.2%	-26.6	14.5	45.3	16	2.83125	3.3016792
LPO-50F	*Canis sp.*	scapula (R)	521	9387.40	9396.90	1.8%	-25.5	14.2	44.2	15.6	2.8333333	3.3041087
LPO-51F	*Canis sp.*	scapula (R)	538	9286.40	9294.20	1.4%	-25.8	15	45.7	16.3	2.803681	3.2695295
LPO-53F	*Canis sp.*	scapula (R)	517	6617.20	6630.20	2.5%	-26.8	15	44.6	15.7	2.8407643	3.3127744
LPO-54F	*Canis sp.*	scapula (R)	524	6682.10	6693.40	2.2%	-26.2	14.7	45.5	15.9	2.8616352	3.3371131
LPO-55F	*Canis sp.*	scapula (R)	531	6629.40	6635.60	1.2%	-27	15.3	43.5	15.1	2.8807947	3.3594561

LPO-57F	*Canis sp.*	scapula (R)	500	6619.00	6631.60	2.5%	-24.9	13.6	44.6	15.6	2.8589744	3.3340102
LPO-58F	*Canis sp.*	scapula (R)	523	9459.80	9466.40	1.3%	-25.5	13.5	45.3	15.8	2.8670886	3.3434727
LPO-59F	*Canis sp.*	scapula (R)	528	9459.20	9469.80	2.0%	-26.7	14.5	43.6	15.4	2.8311688	3.3015846
LPO-62F	*Canis sp.*	scapula (R)	521	6682.70	6688.60	1.1%	-27.1	14	43.9	14.7	2.9863946	3.482602
LPO-63F	*Canis sp.*	scapula (R)	519	6656.90	6671.50	2.8%	-26.1	14.9	42.7	15.1	2.8278146	3.297673
LPO-64F	*Canis sp.*	parietal frag	300	6678.4	6681.50	1.0%	-25.47	12.36	42.73	13.99	3.0543245	3.5618189
LPO-65F	*Canis sp.*	parietal frag	500	6625.9	6634.00	1.6%	-24.65	12.61	44.03	15.89	2.7709251	3.231331
LPO-66F	*Canis sp.*	parietal frag	500	6629	6638.70	1.9%	-25.56	12.47	41.73	14.29	2.9202239	3.4054367
LPO-67F	*Canis sp.*	parietal frag	500	6692.5	6699.70	1.4%	-25.92	13.62	44.38	15.36	2.8893229	3.3694013
LPO-68F	*Canis sp.*	parietal frag	500	6683.80	6691.60	1.6%	-25.1	12.6	45.3	16.1	2.8136646	3.2811719
LPO-70F	*Canis sp.*	parietal frag	500	6612.7	6619.80	1.4%	-25.2	13.2	45.3	16	2.83125	3.3016792
LPO-71F	*Canis sp.*	parietal frag	500	6622.20	6630.50	1.7%	-25.6	13.3	45.1	16	2.81875	3.2871023
LPO-72F	*Canis sp.*	parietal frag	300	6634.5	6638.90	1.5%	-25.09	12.43	44.92	15.78	2.8466413	3.3196279
LPO-73F	*Canis sp.*	parietal frag	500	6641.90	6654.80	2.6%	-25.5	13.5	44.3	15.3	2.8954248	3.3765171

LPO-84F	*Homo sapiens sapiens*	long bone	1003	6666.3	6686.6	2.0%	-26.1	17.3	42.94	14.45	2.9716263	3.4653799
LPO-86F	*Homo sapiens sapiens*	long bone	528.6	6677.8	6687.5	1.8%	-26.2	17	44.15	15.12	2.9199735	3.4051447
LPO-10F	*Lagopus lagopus*	coracoid	300	6714.60	6719.2	1.5%	-21.08	1.53	45.42	15.77	2.8801522	3.3587068
LPO-79F	*Martes zibellina*	tibia	150	6618.2	6619.80	1.1%	-21.98	8.73	42.49	13.87	3.0634463	3.5724563
LPO-11F	*Odobenus rosmarinus*	Ph 1	300	6634.6	6639.20	1.5%	-17.25	12.5	45.4	16.23	2.797289	3.2620754
LPO-25F	*Rangifer tarandus*	talus	450	6638.1	6646.80	1.9%	-19.3	6.93	42.96	15.2	2.8263158	3.2959252
LPO-75F	*Rangifer tarandus*	metacarpus	526	6652.80	6662.50	1.8%	-19.6	8.5	45.9	15.9	2.8867925	3.3664504
LPO-76F	*Rangifer tarandus*	tibia	450	6626.00	6630.60	1.0%	-19.4	3.3	45.8	15.5	2.9548387	3.445803
